# Bacterial Delivery of RNAi Effectors: Transkingdom RNAi

**DOI:** 10.3791/2099

**Published:** 2010-08-18

**Authors:** Hermann Lage, Andrea Krühn

**Affiliations:** Institute of Pathology, Charité Campus Mitte

## Abstract

RNA interference (RNAi) represents a high effective mechanism for specific inhibition of mRNA expression. Besides its potential as a powerful laboratory tool, the RNAi pathway appears to be promising for therapeutic utilization. For development of RNA interference (RNAi)-based therapies, delivery of RNAi-mediating agents to target cells is one of the major obstacles. A novel strategy to overcome this hurdle is transkingdom RNAi (*tk*RNAi). This technology uses non-pathogenic bacteria, e.g. *Escherichia coli*, to produce and deliver therapeutic short hairpin RNA (shRNA) into target cells to induce RNAi. A first-generation *tk*RNAi-mediating vector, TRIP, contains the bacteriophage T7 promoter for expression regulation of a therapeutic shRNA of interest. Furthermore, TRIP has the *Inv* locus from *Yersinia pseudotuberculosis* that encodes invasin, which permits natural noninvasive bacteria to enter β1-integrin-positive mammalian cells and the *HlyA* gene from *Listeria monocytogenes*, which produces listeriolysin O. This enzyme allows the therapeutic shRNA to escape from entry vesicles within the cytoplasm of the target cell. TRIP constructs are introduced into a competent non-pathogenic *Escherichia coli* strain, which encodes T7 RNA polymerase necessary for the T7 promoter-driven synthesis of shRNAs. A well-characterized cancer-associated target molecule for different RNAi strategies is ABCB1 (MDR1/P-glycoprotein, MDR1/P-gp). This ABC-transporter acts as a drug extrusion pump and mediates the "classical" ABCB1-mediated multidrug resistance (MDR) phenotype of human cancer cells which is characterized by a specific cross resistance pattern. Different ABCB1-expressing MDR cancer cells were treated with anti-ABCB1 shRNA expression vector bearing *E. coli*. This procedure resulted in activation of the RNAi pathways within the cancer cells and a considerable down regulation of the ABCB1 encoding mRNA as well as the corresponding drug extrusion pump. Accordingly, drug accumulation was enhanced in the pristine drug-resistant cancer cells and the MDR phenotype was reversed. By means of this model the data provide the proof-of-concept that *tk*RNAi is suitable for modulation of cancer-associated factors, e.g. ABCB1, in human cancer cells.

**Figure:**
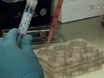


## Protocol

### 1) Bacterial Delivery of shRNAs

1. Before starting with the bacterial culture, one has to prepare LB-medium and LB-agar.
2. For the LB-medium weigh out yeast extract (0.5 % w/v), bacto tryptone (1.0 % w/v), and NaCl (0.6 % w/v) and dilute these components in aqua bidest. The prepared solutions have to be sterilized in an autoclave and are then ready to use.
3. For LB-agar plates bacto agar (1.5 % w/v) has to be added to the LB-medium previous to  sterilization.
4. Heat up LB-agar in the microwave until the LB-agar is completely dissolved. Let the solution cool down until the bottle can be touched easily without getting burned. Add kanamycin (100 mg/ml) for selection of positive clones in further steps.
5. Prepare LB-agar plates at a laminar flow bench (sterile conditions) by pipetting 20 ml of the LB-agar solution into 10-cm Petri-dishes. Let the plates cool down until the fluid has become solid. The plates are now ready to use and can be stored at 4 °C .
6. ShRNA-encoding expression vectors are transformed into competent *E. coli* ceq221 by heat shock using the CaCl_2_ procedure.
7. Plate the transformed bacteria on LB-agar plates containing 100 μg/ml kanamycin, close the lid, seal the plates with  parafilm and cultivate upside down at 37 °C over night.
8. Inoculate bacterial mini cultures with positive clones by picking grown colonies with a tooth pick. Transfer the tooth pick into 7.0 ml of fresh LB-medium containing 100 mg/ml kanamycin and cultivate at 37 °C over night on a shaker at 200 rpm.
9. Inoculate 100 ml of fresh LB-medium containing 100 mg/ml kanamycin 1:100 with the over night mini culture in a 1 l Erlenmeyer flask, and incubate at 37 °C over night on a shaker at 200 rpm.
10. Seed 25 x 10^4^ (number of seeded cells depends on the speed of cell growth of the according cell line; confluency should be ~ 70-80 % 24 hours after seeding) human gastric carcinoma cells (EPG85-257RDB) per well in 6-well dishes and incubate these at 37 °C, in a 5 % CO_2_, water vapor saturated atmosphere over night.
11. Previous to cancer cell infection, over night cultures of *tk*RNAi vector-containing *E. coli* are measured in a photometer to determine the OD_600_. Dilute the bacterial solution until an optical density of 0.5 is reached (OD_600_ = 0.5 equals 1.6 x 10^8^ bacterial cells/ml).
12. Replace the FCS-containing cell culture medium of the carcinoma cells against FCS-free medium 30 min previous to bacterial co-incubation.
13. Wash bacteria twice with 1 x PBS, and dilute in serum-free Leibovitz L-15 medium.
14. Add diluted bacteria to cancer cells at desired MOI (multiplicity of infection = number of bacterial cells per cancer cell) and co-incubate bacteria and cancer cells for 2 hours at 37 °C.
15. After 2 hours of co-incubation cancer cells were washed twice with 1x x PBS and once with serum-containing Leibovitz L-15 medium supplemented with 100 u/ml penicillin, 100 μg/ml streptomycin, 2.5 μg/ml amphotericin, 150 μg/ml gentamicin, and 100 μg/ml kanamycin.
16. Continue cultivating the cancer cells at 37 °C, in a 5 % CO_2_, water vapor saturated atmosphere.
17. The effects of the bacterial treatment can be visualized by fluorescent microscopy, measured by quantitative real-time RT PCR on mRNA level, and by western blot analysis on protein level, and shown with functional assays like FACS analysis, and cell proliferation assays. (if wanted, these procedures can be described in detail).

### 2) Representative Results

If the protocol is performed correctly, the results should be comparable to the ones shown below. 

#### Fluorescence microscopy

Figure 1 shows a human gastric carcinoma cell three hours after bacterial treatment (a) in comparison to an untreated cell (b). Around the nucleus of the treated cell, bacteria can be detected.


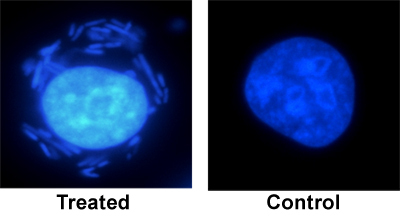
 **Figure 1:** Fluorescent microscopy   Human gastric carcinoma cell after bacterial treatment (1:500) and an untreated human gastric carcinoma cell as control, DAPI-staining, DAPI bandpass filter (Λem = 640 nm), 40x objective .

#### Quantitative real-time RT PCR

In figure 2 a down regulation of MDR1 mRNA of about 70 % (black beam) can be seen after treatment with the therapeutic bacteria. Parental cells serve as a positive control due to the lack of MDR1 overexpression. The cell line 257RDB p170 containing a plasmid expressing anti-MDR1  shRNAs  s taken as direct comparison of the transkingdom RNAi technology to other RNAi silencing strategies. The untreated resistant cell line EPG85-257RDB overexpressing MDR1, the same cell line treated with bacteria lacking the shRNA expressing plasmid, and this cell line treated with therapeutic bacteria carrying a plasmid expressing anti-MRP2 shRNAs were taken as positive controls.


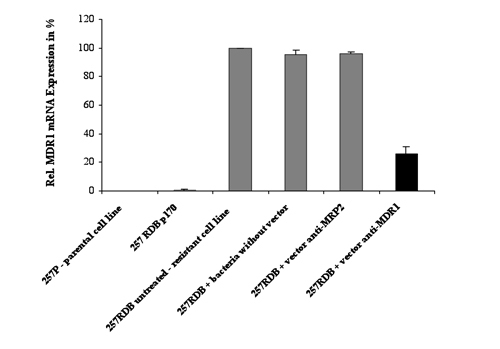
 **Figure 2:** Quantitative real-time RT PCR   MDR1 mRNA expression after treatment with therapeutic *E. coli* ceq221 expressing anti-MDR1 shRNAs (MOI 1 : 500). Normalization was performed using the housekeeping gene aldolase. The MDR1/aldolase ratio of untreated cells of the cell line EPG85-257RDB were set 100 %. P-values were calculated using the student's t-test (* = p<0.05, ** = p<0.005, *** = p<0.001).

**Western blot analysis**

Figure 3 reveals that the MDR1 down regulation also took place on protein level after bacterial treatment. It shows the lacking MDR1 expression of the drug sensitive parental cell line (EPG85-257P), the MDR1 expression of the untreated drug resistant cell line (EPG85-257RDB) and the MDR1 expression of the treated sample. A clear down regulation of MDR1 of the bacterially treated cells can be observed.
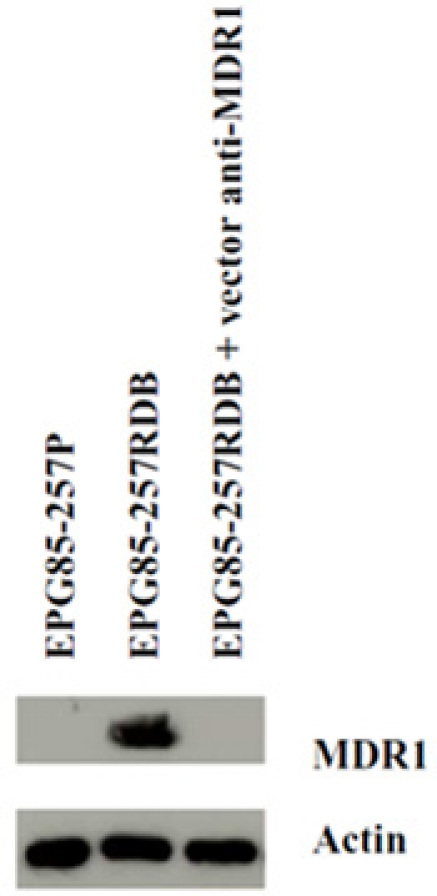


**Figure 3: Western blot analysis.** MDR1 expression levels of the untreated drug-sensitive EPG85-257P, the untreated drug-resistant EPG-257RDB, and of EPG85-257RDB after co-incubation with *E. coli* ceq221 + p43 MDR1. Primary Ab C219 1:100, and anti-actin 1:5 000, secondary Ab anti-mouse 1:10 000.

#### Cytotoxicity assay

Functional analysis like the cytotoxicity assay shown in figure 4 are also indicators for the functioning of the transkingdom RNAi. The parental cell line and the cell line containing an anit-MDR1 shRNA expressing plasmid do not show any resistance to daunorubicin. The resistant cell line EPG85-257RDB and two further controls do not show significant changes in resistance in comparison to the sample treated with anti-MDR1 shRNA expressing bacteria where the resistance to Daunorubicin could be reversed by about 90 %.


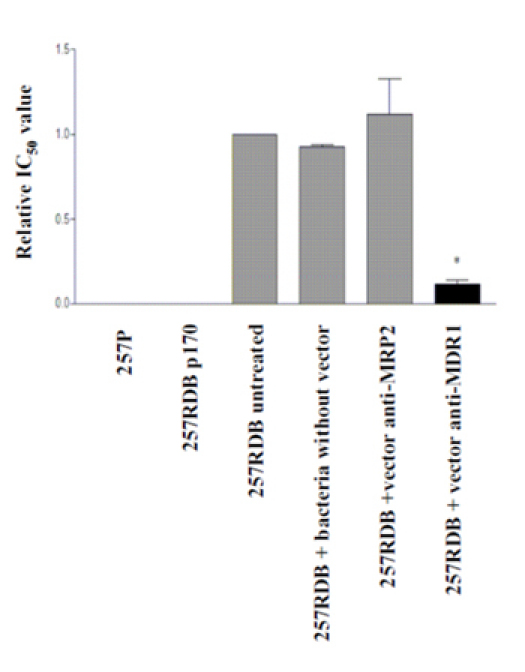
 **Figure 4:** Cytotoxicity assay. Drug-specific IC50-values determined by a cytotoxicity assay for cell survival. P-values were calculated using the student's t-test (* = p<0.05, ** = p<0.005, *** = p<0.001).

#### Anthracycline accumulation assay

According to the lowered resistance of bacterially treated samples (figure 4), the anthracycline accumulation of anti-MDR1 shRNA treated cells is increased by about 90 % as shown in figure 5. The parental cell line and the cell line 257RDB p170 show a strong daunorubicin accumulation up to 100 %. The resistant variant shows rarely any accumulation. Cells treated with anti-MDR1 shRNA expressing bacteria show an anthracyline accumulation increase of 90 %.


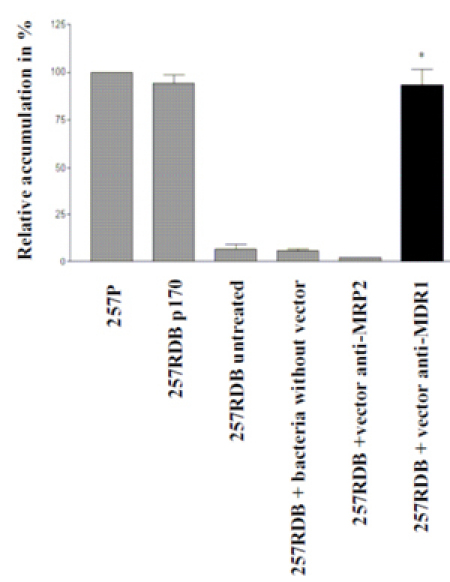
 **Figure 5:** Anthracycline accumulation assay. Anthracycline accumulation of carcinoma cells 6 days after bacterial treatment measured by flow cytometry. P-values were calculated using the student's t-test (* = p<0.05, ** = p<0.005, *** = p<0.001).

## Discussion

The cell number seeded for infection and the corresponding MOI used, critically depend on the cell lines under observation and their speed of growth. To find optimal cell numbers for seeding, pre-experiments to determine the speed of growth are strongly recommended. Besides this, different MOIs should be tested due to the limited extend to which the cells can stand the bacterial invasion without dying of stress. The optimal point of time where the down regulation of the gene under observation may vary. It is recommended to perform experiments over a certain period of time to find the optimal conditions.

## Disclosures

No conflicts of interest declared.
